# FAIR digital twins for biodiversity: enabling data, model, and workflow integration

**DOI:** 10.1038/s44185-025-00116-3

**Published:** 2026-02-02

**Authors:** Sharif Islam, Hanna Koivula, Carrie Andrew, Julian Lopez Gordillo, Claus Weiland, Dmitry Schigel, Dag Endresen, Christos Arvanitidis, Eli Chadwick, Stian Soiland-Reyes

**Affiliations:** 1 Naturalis Biodiversity Center, Leiden, Netherlands; 2 CSC - IT Center for Science, Espoo, Finland; 3 Royal Botanic Gardens, Kew, London, UK; 4 Senckenberg – Leibniz Institution for Biodiversity and Earth System Research, Frankfurt am Main, Germany; 5 Global Biodiversity Information Facility, Copenhagen, Denmark; 6 University of Oslo, Oslo, Norway; 7 LifeWatch ERIC, Seville, Spain; 8 University of Manchester, Manchester, UK

**Keywords:** Ecological modelling, Computational models, Computational platforms and environments, Data acquisition, Data integration, Data processing, Data publication and archiving, Databases, Software, Standards, Statistical methods

## Abstract

The biodiversity crisis demands computational tools to integrate and analyse complex, disparate data and models. This paper presents the concept of FAIR Digital Twins (FDTs) and, drawing on the work of the Biodiversity Digital Twin (BioDT) project (2022–2025), demonstrates how combining Digital Twins with FAIR principles (Findable, Accessible, Interoperable, and Reusable) can transform biodiversity research and decision-making. We show strategies for integrating heterogeneous data, models, and computational workflows within a FAIR framework, paving the way for operational FDTs. The BioDT project developed ten prototype digital twins addressing a critical range of challenges, including grassland and forest dynamics, bird monitoring, ecosystem services, and crop wild relative genetic resources. We discuss implementation challenges such as data fragmentation, semantic interoperability, and operational complexity. Critically, we highlight the opportunities for dynamic adaptation, modular workflows, and cross-domain collaboration, detailing how tools like Research Object Crate (RO-Crate) operationalise FAIR principles for metadata packaging and standardisation. This convergence of Digital Twins with FAIR principles offers a scalable and reusable approach to advancing biodiversity modeling and simulation, providing a robust foundation for evidence-based policy decisions.

## Introduction

The global biodiversity crisis demands multidisciplinary and cross-domain approaches^1^. Progress in this regard is often slowed down by taxonomic, spatial, and temporal data biases, alongside fragmented and disconnected datasets^2–4^. While the FAIR (Findable, Accessible, Interoperable, and Reusable) principles have gained global traction in promoting machine-readable, interoperable, and reusable biodiversity, ecology, and environmental datasets to address some of these challenges^5^, significant gaps still remain when it comes to interoperability and reuse^6,7^. These issues complicate biodiversity monitoring, ecosystem management, and evidence-based policymaking^8,9^. Effective use of modelling is also constrained by heterogeneous data sources, inconsistent protocols, and challenges in reproducing outputs across space and time^10^. Additionally, the success of modelling efforts depends on cross-disciplinary collaboration, as conceptual and terminological barriers often prevent ecologists, biodiversity experts and computer scientists from effectively leveraging one another’s work. Recent advances in generative artificial intelligence further complicate and potentially accelerate these issues^11^. Central to this research and evidence-based policymaking is the integration of heterogeneous datasets and models tailored to diverse use cases^12^. Data may originate from community science platforms, long-term ecological monitoring programmes, natural history museum collections, or specialised repositories focused on particular taxa or domains. And the biodiversity models (whether describing species distributions, population dynamics, interspecies interactions, behavioural patterns, or ecosystem processes) are often developed within the constraints of specific research projects, bounded by time and funding. This focus on methodological innovation is both appropriate and valuable in the research context, yet it presents challenges for long-term reuse and integration in operational services that require models to be consistently maintained, interoperable, and adaptable across varying spatial and temporal scales^13,14^. Dressler et al.^15^ emphasise that upscaling methods in socio-environmental systems must always address heterogeneity. Mrosla et al.^16^, in a systematic literature review, show that modelling flora and fauna in digital twins remains uneven across domains, with gaps in dynamic data availability, model transferability, and metadata standardisation. Similarly, Davison et al.^17^ highlight how fragmented sources and data pipelines require robust validation to enable integration. Together, these studies demonstrate that integrating biodiversity data and models at scale is not merely a question of computational capacity but one of standards, semantics, and shared knowledge.

Within this context, the Digital Twin (DT) paradigm has emerged as a promising method for addressing data and model integration challenges. Proven in fields such as manufacturing and climate modelling, DTs produce dynamic, near real-time simulations that integrate diverse data streams, models, and feedback mechanisms to support decision making^18^. DTs are particularly valuable for studying ecosystems and biodiversity under pressures of global change because the use cases require multiple datasets and models to improve predictive accuracy^19,20^. However, implementing such integrative approaches involves navigating a complex technical landscape, including data standards, modelling frameworks, and ontology design^21^. These complexities are compounded by the imbalance between structured monitoring data and the growing volume of opportunistic and automated observations, both of which challenge the development of robust models for prediction and simulation^22–24^.

The FAIR principles provide a foundation for this integration, applying not only to data but also to software, models, computational workflows, and other digital objects^25^. By combining FAIR and DT frameworks, FAIR Digital Twins (FDTs) extend the DT concept so that different components are machine-actionable, interoperable, and reusable^26^. The modularity inherent in FAIR enables automated operations, cross-domain reuse, trust, provenance tracking and scalable integration. In this sense, FAIR implementation is not an afterthought but a prerequisite for trustworthy and sustainable DTs^27^.

Drawing on experiences from the EU-funded Biodiversity Digital Twin (BioDT) project (2022–2025), this paper shows how FDTs can be operationalised in biodiversity through the integration of heterogeneous data sources and diverse modelling approaches. BioDT developed ten prototype DTs (pDTs) addressing challenges such as grassland and forest dynamics, real-time bird monitoring, ecosystem services, and crop wild relatives for food security. Details of each prototype have been described elsewhere^28^. Here we provide a few examples from these prototypes and focus on the integrative approach: applying FAIR principles to data, models, and workflows.

We showcase the approach using RO-Crate, a lightweight packaging framework for bundling digital objects with machine-readable metadata^29^. Within BioDT, RO-Crate served as the connective tissue linking datasets, models, and workflows across diverse prototypes, ensuring consistency, transparency, and reusability. FAIR principles, implemented through frameworks such as RO-Crate, thus provide the basis for sustainable, interoperable DT infrastructures that bridge fragmented data sources and modelling approaches. See the supplementary information for a glossary of abbreviations and technical terms.

## Results

### RO-Crate profiles

As the BioDT prototypes varied in data sources, granularity, metadata availability, FAIR implementation, and modelling approaches, we needed a strategy that balanced completeness and simplicity. RO-Crate implementation across BioDT’s prototypes enabled machine-readable integration of heterogeneous digital objects. The project successfully packaged datasets, models, workflows, and documentation with provenance tracking, achieving interoperability through Schema.org, Bioschemas.org, and W3C standards. These self-contained packages proved portable across computing infrastructures and reusable across diverse use cases.

**Fig. 1:**
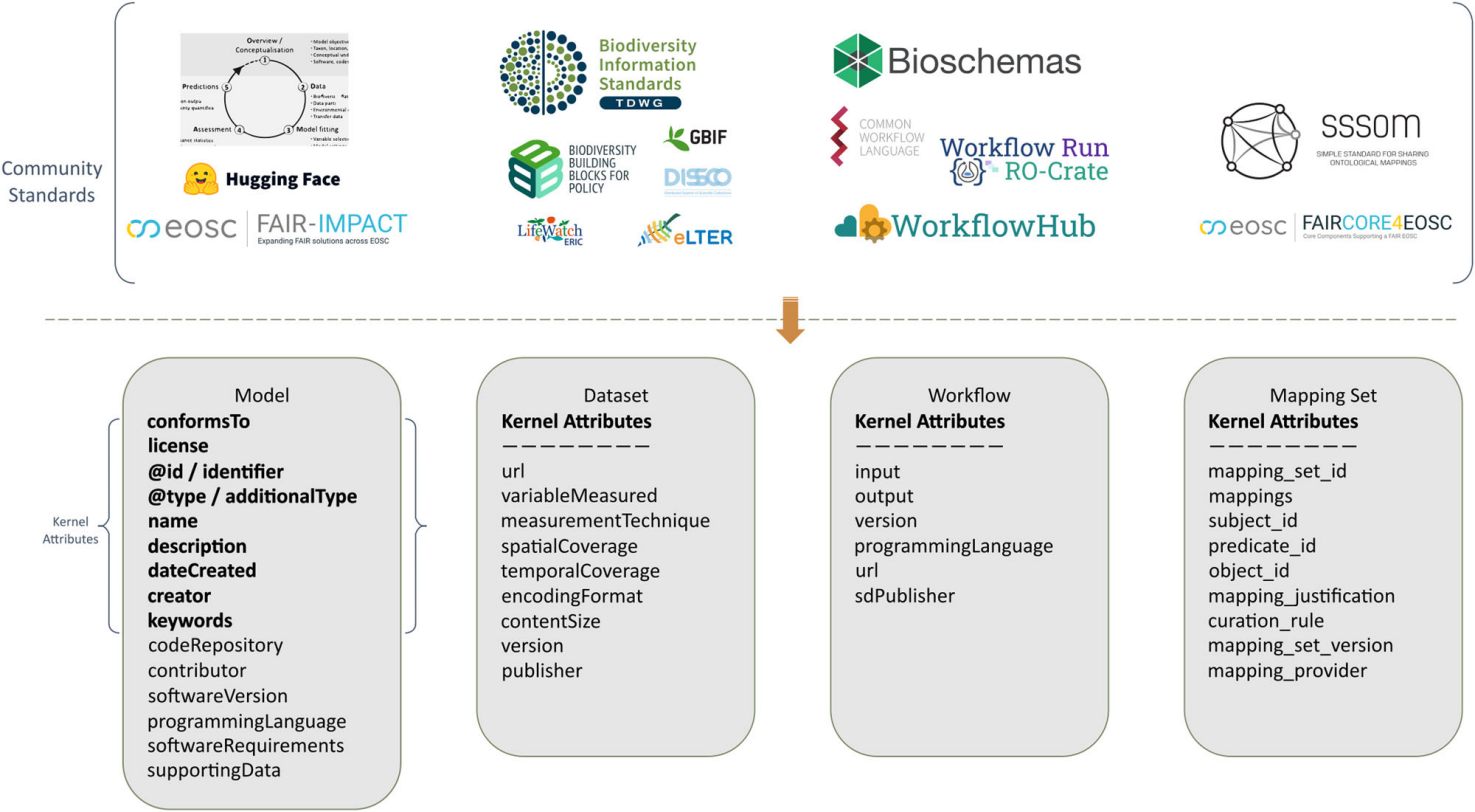
RO-Crate metadata profiles for BioDT. In each of the columns, one of the metadata profiles developed for BioDT is shown. These are: *Model*, *Dataset*, *Workflow*, and *Mapping Set*. The community standards that were taken into account when developing each respective profile is shown above each column (top side of the figure). The metadata attributes that each profile is composed of are listed within each column (bottom side). The kernel attributes are common for all types in BioDT. They are only listed for the Model column (in bold), and are collapsed for the rest of the types.

As part of its FAIR implementation strategy, BioDT developed and tested several RO-Crate profiles (see Fig. 1). These profiles provided enough metadata to support reuse and reproducibility while remaining lightweight. Many properties (e.g., Schema.org/description, Schema.org/spatialCoverage, Schema.org/dateCreated) were already available in Schema.org, and additional terms from community standards (e.g., SSSOM mapping justification, Bioschemas input) were incorporated as needed. The same pattern was applied across models and workflows, where software versions, dependencies, and input/output relationships are recorded to support reproducibility and modular reuse. Because these elements are captured in a unified structure, integration into computational infrastructures such as High Performance Computing systems and cloud environments became significantly easier.

These RO-Crate profiles were deployed across all prototypes. Below, we present three representative examples that illustrate the diversity of data sources, modeling approaches, and integration challenges addressed through this FAIR framework. The pDT web interfaces are currently hosted in a cloud infrastructure supported by LifeWatch ERIC.

### Digital twin prototypes

#### Prototype 1: grassland biodiversity dynamics

The Grassland pDT is built on the individual-based model GRASSMIND, which simulates vegetation dynamics by modelling the establishment, growth, and mortality of individual plants in response to climate, soil, and land management conditions^30^. The pDT integrates input data from Copernicus (ERA5-Land weather), SoilGrids, HiHydroSoil, and local site-level observations, harmonised via reusable scripts and packaged with RO-Crate metadata. A key challenge addressed was the standardisation and calibration of observation data (retrieved from 17 eLTER grassland sites across Europe) to model Plant Functional Type (PFT) dynamics under varying conditions. FAIR integration is achieved through modular workflows, consistent variable transformation routines, and the use of ontologies and taxonomic services (e.g., GBIF taxonomic backbone, TRY traits database) for semantic alignment.

#### Prototype 2: forest biodiversity dynamics

The Forest pDT couples forest landscape simulation (LANDIS-II) with Hierarchical Modelling of Species Communities (HMSC) to assess biodiversity outcomes under alternative forest management and climate change scenarios^31^. It draws on climate data from the Earth System Grid Federation, forest inventory from Finland, land-cover from Copernicus CORINE, and species occurrence data from Finnish Biodiversity Information Facility. Model calibration is informed by trait data and expert knowledge, while FAIR implementation is realised through automated workflows and modular model configurations. Outputs support long-term projections of forest biodiversity under changing conditions, enabling reproducibility and scenario-based decision support.

#### Prototype 3: crop wild relatives and DestinE pilot application

The Crop Wild Relatives (CWR) pDT in BioDT focused on identifying and utilising crop wild relative genetic resources to enhance crop resilience against climate-driven stresses^32^. This pDT was selected for a pilot project within Destination Earth (DestinE), a flagship initiative of the European Commission developing a highly accurate digital model of the Earth. The pilot leveraged DestinE’s advanced capabilities (including management of large-scale Earth observation data and scalable computing infrastructure) to generate habitat suitability maps. Integrating this pDT into DestinE optimises workflows, enhances predictive accuracy, and provides tailored decision-making tools. This synergy demonstrated the scalability and interoperability of pDTs as critical components of broader digital twin ecosystems. As part of the pilot, a migration path for functionally integrating the CWR pDT into the Destination Earth Data Lake (DEDL) was developed in close collaboration with EUMETSAT (The European operational satellite agency for monitoring weather, climate and the environment)^33^.

The integration approach with DestinE builds on the FAIRification of different research objects through two key mechanisms:

1. RO-Crate: Serves as a container representing digital research objects with structured metadata, workflow descriptions, and schema mappings, ensuring cross-domain reusability.
2. FAIR Signposting: Provides machine-interpretable links that map the topology of research objects on the web, defining relationships between components^34^.

**Fig. 2:**
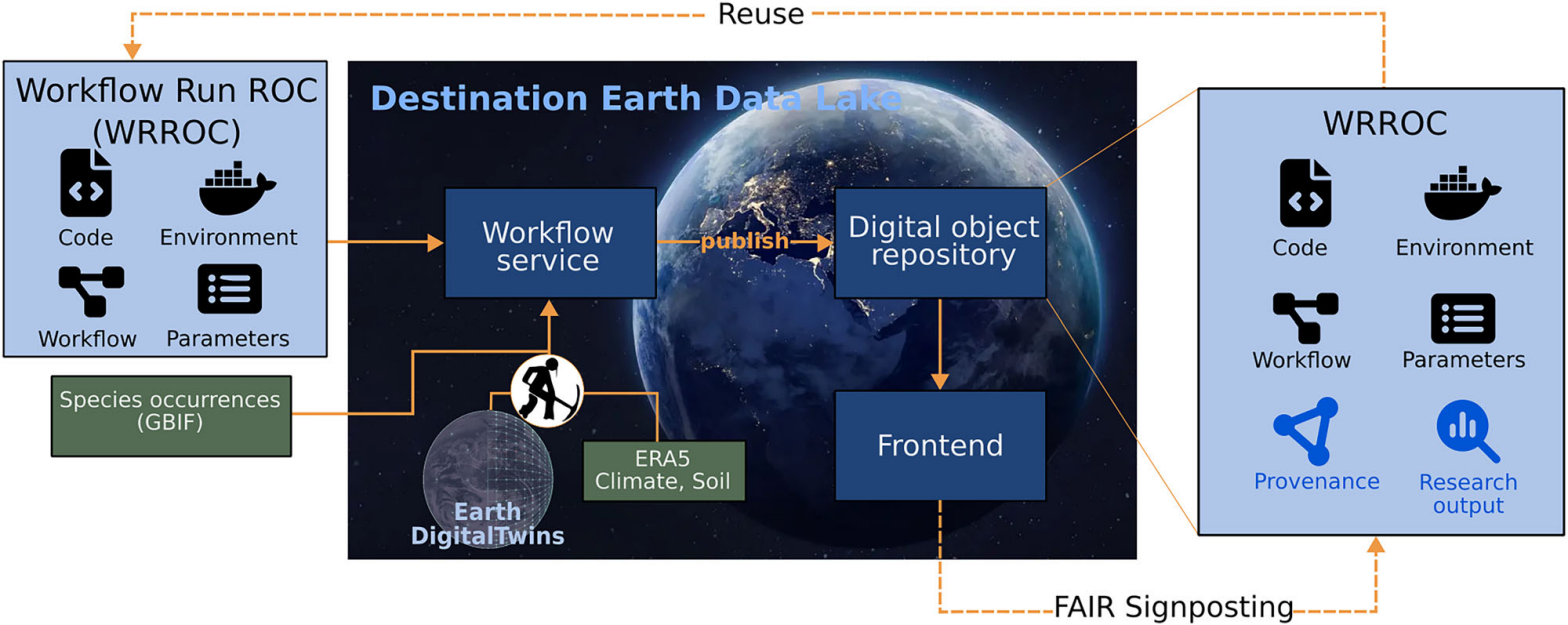
Overview of the Integration of the CWR pDT into the Destination Earth Data Lake (DEDL). The figure illustrates the key components used or implemented in the pilot study, including (i) the data model WRROC, (ii) the workflow service, (iii) the digital object repository, and (iv) a lightweight frontend to mobilize the data using FAIR Signposting. Orchestration with Digital Twins already available in DEDL, such as the Climate Adaptation Digital Twin intended to provide input for the CWR pDT, is currently (as of the time of writing) under implementation. Color code: dark blue: workflow.earth’s service components, light blue: workflow.earth’s data products, green: forcing data.

Using these FAIR components, Workflow Run RO-Crates (WRROC), which is an extension of RO-Crate^35^ can be submitted to DEDL’s workflow service (Fig. 2, middle). A core objective of DEDL is to enable near-data processing (computation executed close to the data source) of DestinE’s comprehensive data portfolio, which includes Copernicus datasets and digital twin outputs. Essential datasets for the CWR pDT, such as ECMWF’s ERA5 soil and climate data, are directly accessible within the data lake, while additional datasets (such as species occurrence data from GBIF) are retrieved via APIs. Workflow execution is facilitated by DEDL’s workflow service (Fig. 2, middle).

After processing, workflow outputs are stored in a digital object repository as reusable WRROCs containing simulation results enriched with detailed provenance information (Fig. 2, right). The relationships among the digital object’s component resources are published through the machine-interpretable FAIR Signposting layer, fostering data discovery and reuse by software agents within and across data spaces.

## Discussion

The BioDT experience demonstrated that FDTs offer a viable pathway for integrating heterogeneous biodiversity data and models into interoperable systems. Through the prototypes, BioDT successfully applied FAIR principles across multiple use cases, established sustainability pathways by transferring services to LifeWatch ERIC, and advanced collaborations with key infrastructures, including GBIF, DiSSCo, eLTER, and LifeWatch ERIC.

The project revealed that FAIR principles serve as both technical enablers and cultural catalysts. Machine-readable metadata, standardised workflows, and provenance tracking ensure data and models remain transparent and usable over time, while RO-Crate demonstrated how harmonised metadata structures can dynamically link models to APIs and different repositories. Critically, provenance and metadata provide the foundation for verifying model accuracy and exposing assumptions in machine-readable formats, enabling both humans and machines to assess DT suitability for specific research questions.

Beyond technical achievements, FAIR and digital twins are driving a cultural transformation in biodiversity science. The convergence of ecologists, data scientists, and digital twin experts is embedding FAIR practices into institutional workflows and community norms. This shift not only supports AI and automation readiness but also increases trust, interpretability, and potential for responsible automation in modelling and decision-support.

Building on insights from other projects on digital twins for ecology and biodiversity^20,36,37^ and drawing on BioDT, we identify key opportunities enabled by this approach. DTs enable near real-time integration of new data, adapting dynamically to changing ecosystem conditions (see Davison et al.^17^ for detailed discussions on this notion of “near real-time” in biodiversity and ecological contexts). Within a FAIR context, this adaptability relates to the findability and accessibility of data streams and the ability of models and tools to facilitate continuous updates. The BioDT project showed that even datasets updated monthly or annually (e.g., soil or vegetation surveys) could be versioned and reused with appropriate metadata, enabling iterative calibration and automation.

DTs are inherently integrative, linking biodiversity use cases with domains such as climate modelling, forestry, and agriculture. The FAIR principles, with their emphasis on interoperability through shared vocabularies, metadata profiles, and persistent identifiers, make such cross-disciplinary collaboration both feasible and scalable. In BioDT, biodiversity occurrence data (e.g., from GBIF) were combined with climate projections (e.g., from Copernicus) and soil data (e.g., SoilGrids), with integration structured through RO-Crate descriptions and harmonised metadata schemas. This approach fosters co-creation and communication across research domains.

The concept of modular construction underpins both FAIR and DT approaches. It concerns the practical implementation of data packages, models, and workflows that can be versioned, reused, and recombined across spatial scales and scientific domains. In BioDT, reusable modules such as weather-soil integration scripts and species distribution modelling workflows were applied across multiple prototype DTs. This scalability reflects broader calls for interoperable building blocks that can be assembled to meet specific ecological or conservation needs^37^.

By linking models with FAIR data and provenance-rich workflows, DTs provide transparent, traceable insights that support evidence-based decision-making. In BioDT, scenarios generated from coupled models (e.g., GRASSMIND or LANDIS-II combined with species distribution models) were grounded in documented inputs and assumptions. Users could trace outputs to specific datasets and parameters. This transparency fosters stakeholder trust and policy uptake. Feedback loops (both human and automated) enable continuous model refinement as new data emerge. Together, these opportunities position DTs as a promising approach to addressing longstanding data and model integration challenges in biodiversity research^37,38^.

However, several challenges emerged during the BioDT project. The BioDT consortium reflected the diversity of data cultures across biodiversity science and ecology. Participants ranged from open data advocates and citizen science communities to ecologists and computer scientists. Many institutions were transitioning from closed practices to open science, FAIR approaches. These differences influenced attitudes toward data publication, reuse, and licensing. Often, scientific outputs were prioritised over infrastructure or metadata development. Fostering a FAIR data culture requires ongoing dialogue, trust-building, and aligned incentives. Although full alignment was not achieved, BioDT initiated conversations and prototyped collaborative strategies that lay the groundwork for systemic shifts.

Biodiversity data are structurally fragmented across global infrastructures. Within BioDT, prototypes combined datasets from multiple sources with varying spatial/temporal resolutions, often differing in format and licensing^21^. Integrating these datasets required substantial manual work. FAIR pipelines based on shared schemas and machine-readable packaging (e.g., via RO-Crate) are thus essential to move from bespoke project-based integration toward reproducible, scalable workflows. Even when datasets are structurally harmonised, differences in conceptual frameworks (e.g., how species, traits, or habitats are defined) pose barriers to integration and interoperability. In BioDT, mismatches in taxonomic resolution, ecological terminology, and provenance granularity led to challenges in model-data integration. These cannot be solved by technical pipelines alone. Semantic alignment requires consistent terms and vocabulary usage within data schemas, along with rich metadata and clear documentation.

Sustaining a FDT framework extends beyond data and models. It requires other components such as computing infrastructure, containerised workflows, metadata indexing, and continuous updates. As Davison et al.^17^ argue, the success of DTs depends on long-term maintenance, not just robust design. In BioDT, transitioning from prototype to operational deployment required significant expertise and coordination. Maintenance often demands more effort than development, yet the biodiversity and ecology domain remains under-resourced in data management and informatics skills^39,40^.

While BioDT’s prototypes prove feasibility, advancing to fully operational FDTs requires addressing fragmented domain-specific standards for occurrence, sequence, trait, and ecological inventory data. Community-driven tools like the GBIF Metabarcoding Toolkit show promise, but need coordinated governance and widespread adoption. Most critically, the field must move beyond self-declared FAIR compliance to automated, scalable validation mechanisms. Efforts and outputs from projects like FAIR-IMPACT and FAIRCORE4EOSC are essential for making FAIRness a verifiable property rather than an aspiration.

The path forward requires sustained investment in governance, training, and policy support across local, national, and global levels. By building on BioDT’s lessons, the biodiversity community can transition from prototype demonstrations to operational infrastructures that are trustworthy, transparent, and capable of supporting both science and policy.

The emphasis on FAIR integration within BioDT offers lessons that extend beyond prototyping digital twins. Collaborations with infrastructures such as GBIF, eLTER, DiSSCo, and LifeWatch ERIC advanced modelling and predictive capabilities, while alignment with the EU’s DestinE Digital Twin Engine demonstrated the potential for FDTs to connect with wider European and global digital ecosystems^41,42^. At the same time, initiatives such as DT-GEO (geophysical extremes), EDITO (European Digital Twin Ocean), and ClimateDT (climate adaptation) highlight the growing momentum behind digital twins across domains, reinforcing the central role of FAIR principles in ensuring interoperability between projects and infrastructures. The BioDT project provided a unique opportunity to bring several components together and create a framework for dialogue with researchers, ecology modellers, and infrastructure and service providers. While the broader solutions to these challenges are outside the scope of this paper, we show that building on the implementation of FAIR principles for digital twins, collaborations with research infrastructure communities, and alignment with global biodiversity data standards offer potential pathways forward.

## Methods

### FAIR implementation in digital twins

Table 1 summarises how each FAIR component was implemented in practice with additional details outlined below. These measures ensured consistent metadata, transparency of provenance, and reusability across diverse datasets, models, and workflows.

**Table 1:** FAIR principles in the context of BioDT implementation

FAIR principles	Key practices in BioDT	Example/notes
Findability	• Use of persistent identifiers (PIDs) for datasets, models, workflows • Metadata-rich RO-Crates with JSON-LD that serve findability • Alignment with community standards (Darwin Core, Schema.org)	• DiSSCo Digital Specimen DOI (as FAIR Digital Object) for museum collection data • GBIF data citation mechanisms via DOI • LifeWatch EcoPortal & Metadata Catalogue • eLTER datasets in B2SHARE • Model metadata in open/public repositories
Accessibility	• Machine-readable terms of access (licensing, usage constraints) • Open APIs for data integration • Hybrid model: public API + HPC internal access • Open data formats • Metadata persistence in registries	• APIs from GBIF, Copernicus, and other infrastructures • Internal HPC access with structured metadata • RO-Crates preserved in B2SHARE and WorkflowHub
Interoperability	• Harmonised metadata and vocabularies • Semantic alignment across standards (Darwin Core, EML, Schema.org) • JSON/JSON-LD enabling compatibility (DCAT, SHACL) • Lightweight Ontology adoption	• Species names harmonised with GBIF Taxonomic Backbone • Pilot semantic mapping service for crosswalks^43^
Reusability	• Provenance-rich metadata and documentation • Modular and versioned workflows • Machine-readable descriptions via RO-Crate • Detailed recording of model dependencies and assumptions	• Grassland pDT: provenance of ERA5-Land, soil, and vegetation integration • Forest pDT: documentation of model linkages and assumptions • Real-Time Bird pDT: versioned RO-Crates for iterative updates^46^

Some of the above points are elaborated below.

### Findability

Each research infrastructure involved in BioDT has already adopted specific FAIR practices to support findability. DiSSCo implemented DOI-based Digital Specimen identifiers; GBIF continued its robust data citation mechanisms; LifeWatch offered EcoPortal and Metadata Catalogue; and eLTER integrated data publishing through B2SHARE. Although BioDT did not provide a unified federated search portal (as datasets and models came from a variety of sources), metadata documentation, indexing and APIs facilitated discovery and internal reuse. Each pDT, for example, was accompanied by an RO-Crate JSON-LD file, which could be deposited in repositories such as B2SHARE (see B2SHARE example from Grassland pDT).

BioDT focused on aligning diverse metadata properties. For example, the RO-Crate for the Grassland pDT includes PIDs for contributors (ORCIDs), links to version-controlled repositories, spatial and temporal coverage information, licensing information, and specific software dependencies. Thus, providing a rich, traceable metadata record.

To support scalable implementation, BioDT developed a suite of profiles defining a set of attributes for datasets, models, workflows, and mappings (see Fig. 1). Community-supported standards (such as Darwin Core and Schema.org) were also essential for ensuring consistency and integration across RIs. To further support findability and semantic interoperability, a pilot semantic mapping service was developed to create and register mappings as FAIR Digital Objects^43^. These mappings serve as lightweight crosswalks between terms that were applied to different pDTs. External registries like the EOSC Metadata Schema and Crosswalk Registry (MSCR) may further enhance such mappings once these services mature.

Despite these tools, metadata adoption posed a steep learning curve for many contributors. Differences in domain conventions, tooling familiarity, and infrastructure support meant that documentation and training were critical. As Ingenloff^44^ highlights in the context of integrating eLTER data into the Grassland pDT, community standards such as Darwin Core and aggregators like GBIF provide practical, complementary mechanisms to improve FAIRness and hence provide context for the data to improve transparency and reproducibility of the pDT. This would allow potential re-users of the model to evaluate if the model is suitable to be used outside of the original context.

### Accessibility

BioDT adopted a dual strategy for access:

- Use of open APIs enabled access to datasets for integration with workflow tools. These APIs are provided by the researcher infrastructures and various data providers.
- For internal High Performance Computing users, datasets were made accessible within internal file systems paired with structured metadata and internal indexing to uphold FAIR standards.

This hybrid model ensures that data remains accessible in both public and internal computing environments. Metadata profiles and APIs further support automated retrieval and cross-platform access.

Using open, non-proprietary formats is key to ensuring accessibility and longevity. BioDT implemented RO-Crate containers based on open standards (e.g., JSON-LD, Schema.org, Darwin Core), ensuring that metadata and resources remain portable across platforms.

BioDT RO-Crate files have been preserved in sustainable registries such as B2SHARE, WorkflowHub, ensuring long-term accessibility even if the data and models themselves become unavailable. This aligns with FAIR goals of metadata persistence and traceability^45^.

### Interoperability

Cross-domain interoperability depends on aligning with widely adopted standards such as Darwin Core, EML, and Schema.org. BioDT collaborated with TDWG and EOSC initiatives to adopt shared ontologies and vocabularies. For example, species names were harmonised using the GBIF taxonomic backbone. Metadata profiles also drew on existing templates, enabling cross-platform reuse.

Semantic alignment is essential for integrating datasets from sources like GBIF, remote sensing, and national repositories (e.g., https://laji.fi/en). BioDT employed JSON and JSON-LD serialisation formats, enabling compatibility with DCAT and SHACL specifications. This flexibility allows seamless linking of data, models, and workflows across multiple DTs, ensuring consistent interpretation and reusability.

### Reusability

RO-Crate enabled BioDT to capture detailed provenance, including software environment, library dependencies, and processing history. In the Grassland pDT, for instance, provenance metadata documents how ERA5-Land weather data was interpolated and harmonised with soil and vegetation datasets, ensuring reproducibility and model calibration^30^. Similarly, for the Forest Biodiversity pDT, model interlinkages and assumptions are recorded for traceability^31^.

BioDT’s modular workflow design allows components to be reused and updated independently. For example, in the Real-Time Bird Monitoring pDT, iterative updates to species distribution models are documented through versioned RO-Crates, supporting historical comparisons and reproducibility^46^. This modularity enables workflows (e.g., soil data pipelines or species distribution tools) to be reused across use cases, such as forest or pollinator modelling, demonstrating scalable design.

RO-Crate provides a consistent structure for describing relationships among datasets, models, workflows, and outputs. This supports integration with APIs, semantic validators, and other DTs.

Building on the FAIR principles and the RO-Crate profiles described in the Results section, we now detail how these profiles were applied to specific infrastructural and operational components. These components (datasets, models, and workflows) address the complexity and dynamic nature of biodiversity data, models, and applications, forming the building blocks of FDTs.

### RO-crate components

The dataset component of a DT captures the primary input needed for analysis or modelling. A key example is the Grassland dynamics pDT dataset from the eLTER Zone Atelier Armorique. This dataset is documented with metadata including creator ORCIDs, publication dates, licensing terms, spatial and temporal coverage, and PIDs for associated supporting data (see a working example of a dataset RO-Crate in GitHub and final published RO-Crate deposited in the B2SHARE repository). This enables reuse across applications such as ecosystem service assessment or species distribution modelling.

Metadata for models demonstrates how DTs can integrate computational tools to derive insights, such as mapping species distributions or evaluating ecosystem services. The RO-Crate example for the biodiversity model in the Cultural Ecosystem Services pDT illustrates how to represent and share computational models. This specific RO-Crate documents a species distribution model designed to analyse species contributing to cultural ecosystem services, such as recreation and aesthetic values. Key metadata includes links to supporting datasets, the model’s version, its programming environment (R), and software dependencies. The RO-Crate also references the GBIF dataset (with DOI) used for occurrence records and environmental filtering, providing transparency and traceability for provenance and inputs.

The workflow component connects datasets and models into cohesive analytical pipelines. For example, the metadata for the ModGP (Modelling the Germplasm of Interest) workflow from the Crop Wild Relative pDT documents the steps for data preparation, model execution, and output generation^32^. These steps (also in other pDTs) often can be further broken into granularity, such as downloading GBIF data, executing scripts, and validating results. The metadata for the steps is thus essential for reproducibility and provenance tracking. Although not all steps were fully automated within BioDT, the structure and links are in place to support future development. One scalable method to organise this metadata that was used is by adopting the Bioschemas ComputationalWorkflow profile. Reusing these profiles, BioDT created workflow descriptions (capturing execution environments, inputs, outputs, and provenance of computational runs, etc.) that can be readily integrated into other digital twin infrastructures.

### Bridging FAIR principles and DT components

Operational infrastructure is essential for collecting, harmonising, processing, and automating diverse data streams from sources such as automated sensors, human observations, historical datasets, and environmental records. For example, BioDT’s Forest Dynamics and Real-Time Bird Monitoring pDTs integrate climate projections, forest inventories, and bird occurrence data to simulate long-term dynamics. Ensuring interoperability between such heterogeneous data sources requires tools like APIs, semantic standards, and frameworks such as RO-Crate. When packaged with RO-Crate, these datasets and metadata become portable and interoperable, ready to be processed and managed on different compute infrastructures as needed.

In BioDT, models ranged from mechanistic simulations, such as agent-based models, to statistical approaches, such as species distribution models^23^. Mechanisms for calibrating models with real-world observations and historical data, as seen in the Grassland Biodiversity Dynamics pDT, ensure continuous improvement in predictive accuracy. BioDT’s ability to leverage the computational power of HPC infrastructures like LUMI ensures that even complex, hybrid models can be efficiently run and iteratively improved.

An important aspect of FDT is not only integration but also transparency about uncertainty, scope, and validation. Models differ in predictive reliability depending on their design and data inputs: correlative SDMs are powerful but can struggle when extrapolating to novel conditions, whereas mechanistic or agent-based models incorporate causal processes but are often limited by data availability and parameterization requirements^47^. By combining process-based models with heterogeneous data sources, the DT framework can enable systematic testing of predictive capabilities across gradients of complexity, from controlled case studies to spatially extensive, multi-ecosystem systems.

Within each pDT, FAIR metadata (capturing provenance, assumptions, and parameter choices) makes explicit the valid use cases and limitations of the model. This helps researchers and stakeholders assess whether a DT is suitable for a given question and reduces the risk of out-of-scope applications. Moreover, versioned RO-Crates documenting inputs, assumptions, and outputs provide an avenue for extending DTs with new datasets, while ensuring that such integrations remain traceable and transparent. In this way, our approach in BioDT not only integrated data and models but also embedded mechanisms for communicating uncertainty, scope, and reusability to end-users.

Unlike industrial settings, such as car manufacturing, where feedback loops are tightly coupled with sensor-driven automation and control systems, feedback in biodiversity contexts often relies on asynchronous, heterogeneous inputs from field observations, institutional updates, and community contributions. While less continuous or automated, these feedback processes can still support dynamic refinement of models and data. BioDT pDTs demonstrate how adapting FAIR principles enables meaningful synchronisation between digital representations and evolving ecological realities.

To be impactful, DTs must translate complex simulations into actionable insights for stakeholders. For instance, BioDT created a R-Shiny visualisation tool (https://app.biodt.lifewatch.eu/; source code: https://github.com/BioDT/biodt-shiny) that enables end-users to explore and interact with dynamic models through user-friendly interfaces. These cloud-based tools foster a collaborative and informed approach to biodiversity management by making complex model outputs accessible and interpretable.

By integrating FAIR principles with practical tools and frameworks, BioDT provides a blueprint for scalable, interoperable, and sustainable DTs in biodiversity research.

Packaging data and workflows with RO-Crate ensures compatibility with infrastructures like LUMI and other cloud platforms, enabling efficient visualisation and decision-support tools for end-users. As McInerny et al.^48^ argue, ecology and biodiversity research have long lacked sufficient expertise, skills, and institutional capacity in scientific visualisation, despite its central role in communicating complexity. The issue is not only how to embed visualisation expertise within scientific organisations, but also how to provide training and sustainable support. Dobson et al.^49^ further emphasise that the greater the sophistication of an analysis, the harder it becomes to summarise for non-specialist audiences. Different audiences may require different modes of visualisation, and communicating uncertainty about data and models in an accessible way remains a major challenge.

BioDT addressed these gaps through a dedicated work package on user requirements, service uptake, and training, where FAIR and Open Science principles were explicitly integrated. Visualisation was developed in close collaboration with each pDT team to ensure relevance to user needs. The R-Shiny interfaces, for example, were designed with these considerations in mind, balancing complexity with accessibility. Sustainability was also built into the plan: LifeWatch ERIC has committed to hosting and maintaining the R Shiny services beyond the project lifetime. In parallel, BioDT organised a hackathon school and produced training materials to build user capacity and strengthen community practices, helping to ensure that visualisation and user interface remain a living, evolving component of FDTs.

**Fig. 3:**
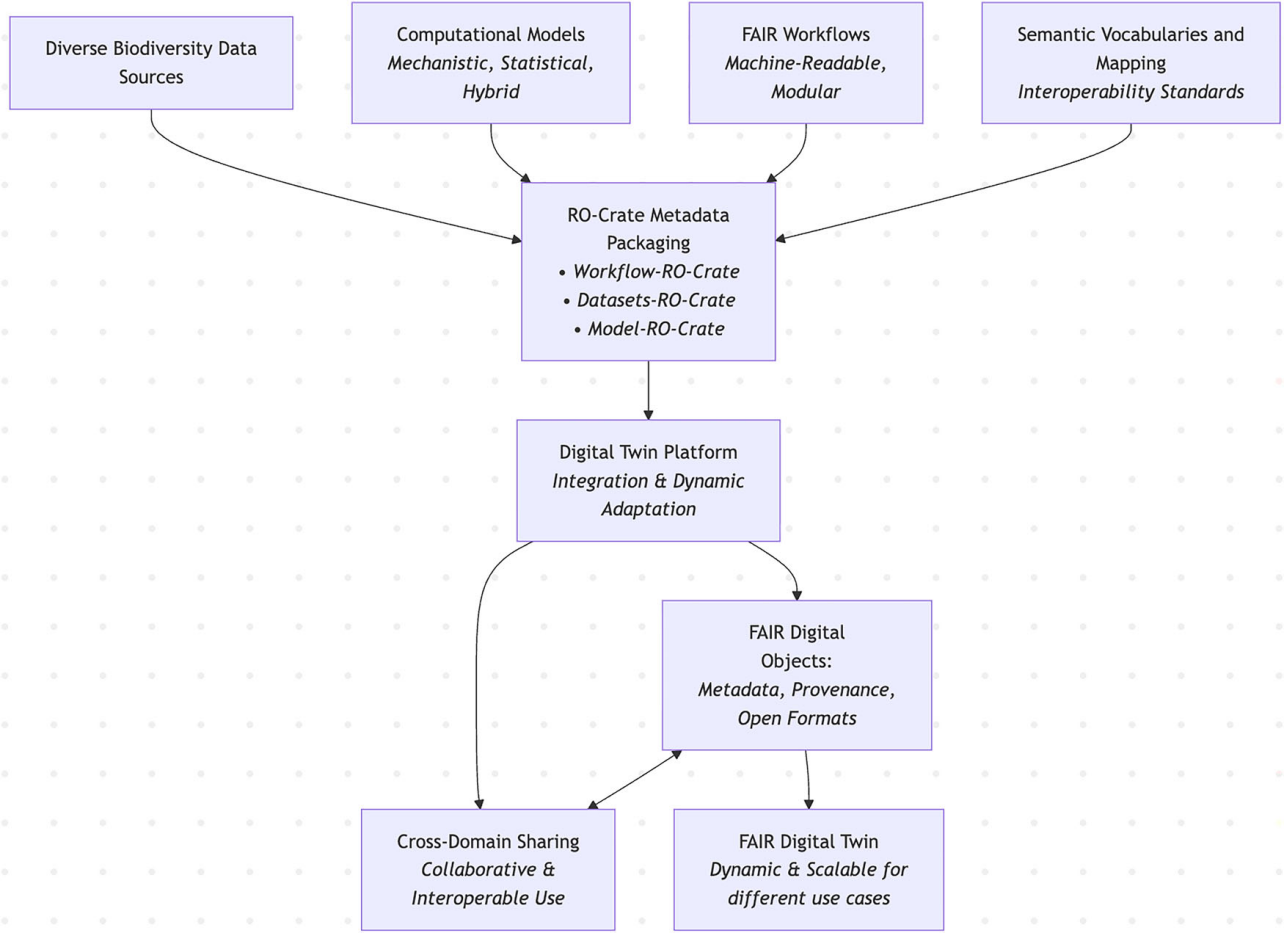
A simple schematic to show how the FAIR Digital Twin and RO-Crate integrate components.

The RO-Crates described in the Results section collectively form the building blocks towards FDT implementation (see Fig. 3 for a simple schematic describing RO-Crate integrating the components). Together, they enable a scalable system adhering to FAIR principles:

- Findability: Persistent identifiers and rich metadata make datasets, models, and workflows easily discoverable. These PIDs from different sources are described and linked in each RO-Crate in a machine-readable format.
- Accessibility: RO-Crate, as well as RO-Crate profiles, use JSON-LD, which is a standard way to serialise content. If another open, interoperable serialisation method is preferred, that could be easily adopted.
- Interoperability: Use of common vocabularies and profiles within RO-Crate, such as Schema.org and Bioschemas, supports integration with existing infrastructures. These profiles can be customised and extended with domain-specific standards.
- Reusability: Detailed provenance, versioning, and adherence to community-recognised standards ensure reproducibility and adaptability. The computational workflow component plays an important role here. The Workflow Run Crate extension captures comprehensive execution provenance of computational workflows, bundling inputs, outputs, and code while supporting different granularity levels. This approach enables interoperable comparisons between workflow runs from heterogeneous systems and aligns with standards such as W3C PROV^35^.

## Supplementary information

Supplementary Information

## Data Availability

The datasets used in this study originated from various sources and were integrated across the ten BioDT prototypes. Detailed documentation of individual datasets is available in the Research Ideas and Outcomes collection papers (10.3897/rio.coll.240). Selected datasets have been deposited in B2SHARE (https://b2share.eudat.eu/records/?q=BioDT). RO-Crate metadata packages are available in WorkflowHub (https://workflowhub.eu/programmes/22#projects) and GitHub (https://github.com/BioDT/biodt-fair). Example RO-Crates include the Grassland pDT dataset (http://hdl.handle.net/11304/f1a6c91f-e9b6-4b16-b219-433d5fb87f64) and the Cultural Ecosystem Services biodiversity model (https://github.com/BioDT/biodt-fair/blob/main/pDTs/ces/ces_biodiversity_model_ro-crate.json).

## Code availability

All scripts for generating RO-Crate metadata are available in the BioDT GitHub organisation (https://github.com/BioDT). The R Shiny visualisation tool for exploring prototype outputs is accessible at https://app.biodt.lifewatch.eu/, with source code available at https://github.com/BioDT/biodt-shiny. Individual prototype workflows and code are documented in their respective GitHub repositories within the BioDT organisation.
