# Supplementary material for: FAIR digital twins for biodiversity: enabling data, model, and workflow integration
Source: NPJ Biodivers. 2026 Feb 2;5:5. doi: 10.1038/s44185-025-00116-3 (PMC12864969; doi:10.1038/s44185-025-00116-3)
Supplement: Supplementary file 1 — Supplementary Information [file 44185_2025_116_MOESM1_ESM.pdf]

Supplementary Information

Supplementary Table 1: A list of glossary

Abbreviation/Term	Full Term	Explanation
API	Application Programming Interface	A set of protocols and tools for building software applications that allows different programs to communicate with each other
BioDT	Biodiversity Digital Twin	EU-funded project (2022-2025) developing prototype digital twins for advanced modelling, simulation and prediction capabilities for biodiversity research and policy. https://doi.org/10.3030/101057437
CES	Cultural Ecosystem Services	Cultural ecosystem services refer to non-material benefits people obtain from ecosystems, including recreation, tourism, intellectual development, spiritual enrichment, reflection and aesthetic experiences. BioDT created a prototype digital twin for this (see https://doi.org/10.3897/rio.10.e125450)
CWR	Crop Wild Relatives	CWR are wild plant species closely related to cultivated crops. Broadly, they encompass all wild plants within the same genus as the crop. BioDT created a prototype digital twin for this (see https://doi.org/10.3897/rio.10.e125192)
Darwin Core	-	A standard for biodiversity data that provides a stable, straightforward and flexible framework for sharing information about the geographical and temporal occurrence of species
DCAT	Data Catalog Vocabulary	W3C standard vocabulary for describing datasets in data catalogs
DEDL	Destination Earth Data Lake	Data lake enabling access and processing of DestinE's comprehensive data portfolio including Copernicus datasets and digital twin outputs

DestinE	Destination Earth	Flagship initiative of the European Commission to develop a highly-accurate digital model of the Earth
DiSSCo	Distributed System of Scientific Collections	European research infrastructure for natural science collections
DT	Digital Twin	Dynamic, near real-time simulation that integrates diverse data streams, models, and feedback mechanisms to support data-driven decision making
EOSC	European Open Science Cloud	European initiative to provide researchers with access to data, tools, and services across disciplines and borders
ERA5-Land	ECMWF Reanalysis v5	ERA5 is the fifth generation ECMWF atmospheric reanalysis of the global climate covering the period from January 1940 to present. ERA5 is produced by the Copernicus Climate Change Service (C3S) at European Centre for Medium-Range Weather Forecasts (ECMWF).
FAIR	Findable, Accessible, Interoperable, Reusable	Principles for making data and other digital objects machine-actionable and reusable. More information: https://www.go-fair.org/
FDT	FAIR Digital Twin	Digital twin that applies FAIR principles to ensure components (data, models, workflows, metadata) are machine-actionable, interoperable, and reusable.
GBIF	Global Biodiversity Information Facility	International data aggregator providing open access to biodiversity data. https://www.gbif.org/
HMSC	Hierarchical Modelling of Species Communities	HMSC employs generalised linear models to analyse how species respond to environmental conditions, leveraging shared information across species based on their trait similarities and phylogenetic relationships. The model

		combines species trait data with occurrence records to project how forest management practices and climate change may affect biodiversity. LANDIS-II outputs, including projected tree biomass and age structure, are used as environmental predictors in HMSC to generate species distribution projections.
HPC	High Performance Computing	Use of supercomputers and parallel processing techniques for solving complex computational problems
JSON-LD	JavaScript Object Notation for Linked Data	Method of encoding linked data using JSON format
LANDIS-II	-	Forest landscape simulation model used to assess biodiversity outcomes under alternative management and climate scenarios https://www.landis-ii.org/
LUMI	Large Unified Modern Infrastructure	European supercomputer infrastructure. https://www.lumi-supercomputer.eu/
MSCR	Metadata Schema and Crosswalk Registry	EOSC service for managing metadata schemas and their mappings
pDT	Prototype Digital Twin	Experimental or demonstration version of a digital twin developed within the BioDT project
PFT	Plant Functional Type	Classification of plants based on their functional characteristics rather than taxonomic relationships
PID	Persistent Identifier	Long-lasting reference to a digital object that remains active over time
RDF	Resource Description Framework	W3C standard model for data interchange on the web

RO-Crate	Research Object Crate	Lightweight packaging framework for bundling digital objects with machine-actionable metadata using JSON-LD
Schema.org	-	Community effort to create, maintain, and promote structured data schemas for the internet
SHACL	Shapes Constraint Language	W3C standard for validating RDF graphs against constraints
TRY	-	Global database of plant traits. The TRY initiative and database is hosted, developed and maintained at the Max Planck Institute for Biogeochemistry in collaboration with the German Centre for Integrative Biodiversity Research (iDiv) Halle-Jena-Leipzig. https://www.try-db.org/TryWeb/Home.php
WRROC	Workflow Run RO-Crate	RO-Crate profile specifically designed to capture comprehensive execution provenance of computational workflows
eLTER	European Long-Term Ecosystem Research	Research infrastructure providing access to long-term ecosystem research data and services
